# Characterization of chronic relapsing antibody mediated arthritis in mice with a mutation in *Ncf1* causing reduced oxidative burst

**DOI:** 10.1186/s43556-022-00076-1

**Published:** 2022-05-12

**Authors:** Peibin Liang, Yanpeng Li, Rui Xu, Kutty Selva Nandakumar, Roma Stawikowska, Gregg B. Fields, Rikard Holmdahl

**Affiliations:** 1 Medical Inflammation Research, Pharmacology School, Southern Medical University, Guangzhou, 510515 China; 2 Medical Inflammation Research, Department of Biochemistry and Biophysics, Karolinska Institute, SE-17177 Stockholm, Sweden; 3 Department of Chemistry & Biochemistry and I-HEALTH, Florida Atlantic University, Jupiter, FL USA

**Keywords:** Rheumatoid arthritis, Anti-COL2 antibody, Collagen type II induced arthritis, Neutrophil cytosolic factor 1, Chronic phase

## Abstract

Rheumatoid arthritis (RA) is a chronic autoimmune disorder affecting joints with a hallmark of autoantibody production. Mannan-enhanced collagen type II (COL2) antibody induced arthritis (mCAIA) in neutrophil cytosolic factor 1(*Ncf1*) mutation mouse is a chronic disease model imitating RA in mice. In this study, we characterize the chronic phase of mCAIA in *Ncf1* mutated (BQ.*Ncf1*^m1j/m1j^) mice. Arthritis was induced by an intravenous injection of anti-COL2 monoclonal antibodies on day 0 followed by intra-peritoneal injections of mannan (from *Saccharomyces cerevisiae*) on days 3 and 65 in BQ.*Ncf1* ^m1j/m1j^ and BQ mice. Bone erosion was analysed by computed tomography (CT) and blood cell phenotypes by flow cytometry. Cytokines and anti-COL2 antibodies were analyzed with multiplex bead-based assays. The arthritis in the *Ncf1*^m1j/m1j^ mice developed with a chronic and relapsing disease course, which was followed for 200 days and bone erosions of articular joints were evaluated. An increased number of circulating CD11b^+^ Ly6G^+^ neutrophils were observed during the chronic phase, together with a higher level of G-CSF (granulocyte colony-stimulating factor) and TNF-α. In conclusion, the chronic relapsing arthritis of mCAIA in the *Ncf1*^m1j/m1j^ mice develop bone erosions associated with a sustained neutrophil type of inflammatory responses.

## Introduction

Rheumatoid arthritis (RA) is a chronic autoimmune disorder, characterized by joint inflammation, bone destruction and joint pain [1]. The disease is strongly associated with certain major histocompatibility complex haplotypes, indicating an important role for T cells [2, 3]. The presence of autoreactive antibodies in blood is a hallmark of the disease. Antibodies to immunoglobulin (rheumatoid factors, RF) and to citrullinated proteins (anti-citrullinated protein antibodies, ACPA) can be detected years before the clinical onset and are sustained during the chronic disease development [4–6]. Inflammation is mainly affecting the peripheral cartilaginous joints but antibodies to cartilage proteins, such as type II collagen (COL2), are detectable for the first time around the onset of arthritis [7, 8] and are more enriched in the synovial fluid compared to blood [9]. Anti-COL2 antibodies are pathogenic, as observed in the collagen antibody induced arthritis (CAIA) animal model [10–13].

RA is a chronic disease preceded by three distinct phases [14]. The disease process is likely initiated by autoimmune priming leading to increased levels of antibodies to citrullinated proteins and rheumatoid factors in blood, followed several years later by an onset of joint inflammation and the development of a chronic relapsing arthritis characterized by inflammatory bone erosions and joint deformity. The typical CAIA model, induced in mice with or without injection of LPS (Lipopolysaccharide) [15], is a largely acute animal model with a disease course limited to 3–4 weeks. We recently reported a new variant of the CAIA model, induced with anti-COL2 antibodies and an intraperitoneal (i.p.) injection of mannan, in neutrophil cytosolic factor 1 (*Ncf1*) mutated mice (*Ncf1* ^m1j/m1j^), named mannan-enhanced CAIA (mCAIA) [16].

The *Ncf1* protein plays a critical role in the generation of NADPH oxidase 2 (NOX2) complex, the main inducer of reactive oxygen species (ROS). Polymorphisms in the *Ncf1* gene, leading to a deficient ROS response, has been shown to be a major genetic factor in both experimental [17, 18] and human chronic autoimmune diseases [19–21].

NOX2 released ROS plays a regulatory role in chronic autoimmune disease but it may operate through several different mechanisms [22]. The CAIA model is for example regulated by *Ncf1* differently at various phases, which depend on how the disease is enhanced by a secondary injection of adjuvant [23]. Interestingly, the enhancement of arthritis induced by LPS in the CAIA model is counteracted by the *Ncf1* mutation [23], but is more severe if enhanced by mannan [16]. We have earlier shown that mCAIA is not dependent on the activation of the adaptive immune system (T and B cells) and does not require FcγRIII but is instead driven by macrophages deficient in ROS production [16]. Herein, we further investigate the mCAIA model whether ongoing active inflammation and destruction of joints occur during the chronic phase, similar to RA.

## Results

### Induction of mCAIA

**Fig. 1:**
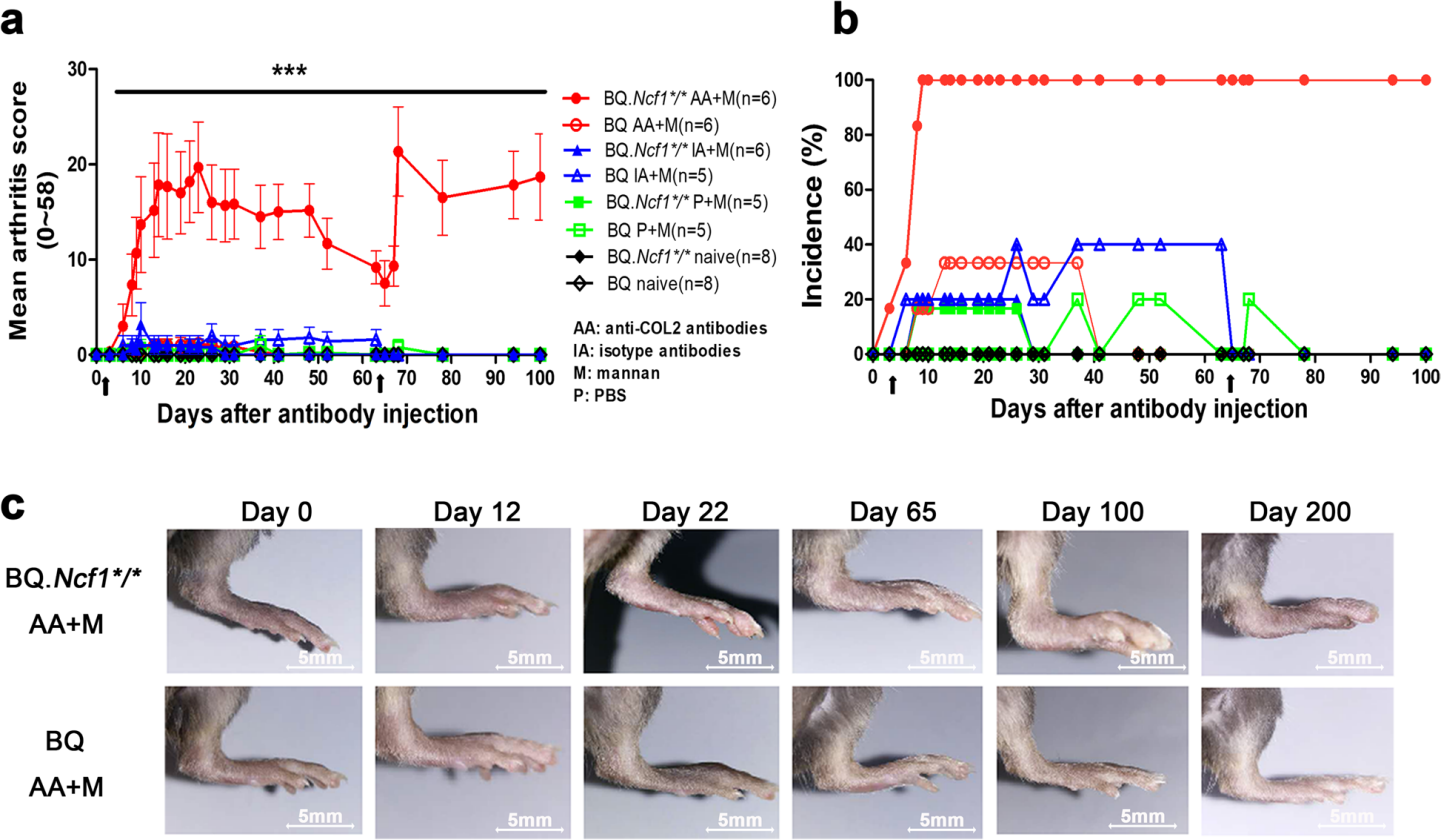
Chronic arthritis developed in the BQ.Ncf1^*/*^ mice after injection of anti-COL2 antibodies and mannan. BQ.Ncf1^*/*^ mice and BQ mice were i.v. injected with anti-COL2 antibody cocktail containing M2139 + UL1 + ACC1 (3 mg each) or the isotype antibody cocktail of G11 (6 mg) + L243 (3 mg) as a control on day 0 and 4 mg of mannan was injected i.p. on days 3 and 65. (In this project, we injected anti-COL2 antibodies into 10 BQ.Ncf1^*/*^ mice and 8 BQ mice in total. For other groups, *n* = 5–8. **a** Arthritis score, (**b**) Disease incidence and (**c**) Ankle morphology in the anti-COL2 antibody cocktail injected BQ.Ncf1^*/*^ mice and BQ mice. Black arrows indicate i.p. injection of 4 mg of mannan each time per mouse, total 2 times. The two-tailed Mann-Whitney U test was used to calculate the level of significance. BQ.Ncf1^*/*^ AA+M vs BQ AA+M: ***, *p* <  0.001. Values shown are mean ± SEM

RA is a chronic inflammatory disease affecting humans. To resemble RA, the mouse model developed should have a long-term and severe arthritis phenotype. Therefore, in this study, mCAIA with a chronic disease induced by anti-COL2 antibodies and mannan was selected and characterized. To induce mCAIA, monoclonal antibodies were purified, quantified, sterilized, and the concentrations were determined (Table 1). BQ.Ncf1^*/*^ and BQ mice were i.v. injected with the monoclonal antibody cocktail M2139 + UL1 + ACC1 (1:1:1, 9 mg total IgG) to induce mCAIA on day 0. As a control, G11 + L243 (2:1, 9 mg total IgG) were injected into a separate group of mice. Mannan was i.p. injected on days 3 and 65. After mCAIA induction, arthritis score and incidence were recorded all along the experiment. BQ.Ncf1^*/*^ mice, but not BQ mice, developed an early onset of severe arthritis with a high disease incidence followed by a chronic and relapsing disease course [Fig. 1a and b]. The affected joints had classical signs of arthritis such as redness, swelling and deformity [Fig. 1c]. The data showed that we successfully established the mCAIA with chronic phase with severe arthritis in *Ncf1* mutated mice.

**Table 1:** Total IgG and anti-COL2 specific IgG concentrations after purification from mouse ascitic fluid

Clone	Anti-COL2 epitope	Subtype	Anti-COL2 specific IgG (mg/ml)	Total IgG (mg/ml)	Ratio (anti-COL2 specific IgG/total IgG)
M2139	J1	IgG2bκ	2.2	10.7	0.2
UL1	U1	IgG2bκ	4.8	24.9	0.2
ACC1	C1	IgG2a	3.5	7.7	0.5
G11	/	IgG2bκ	/	8.3	/
L243	/	IgG2a	/	12.3	/

### Bone erosions in the chronic phase of mCAIA

**Fig. 2:**
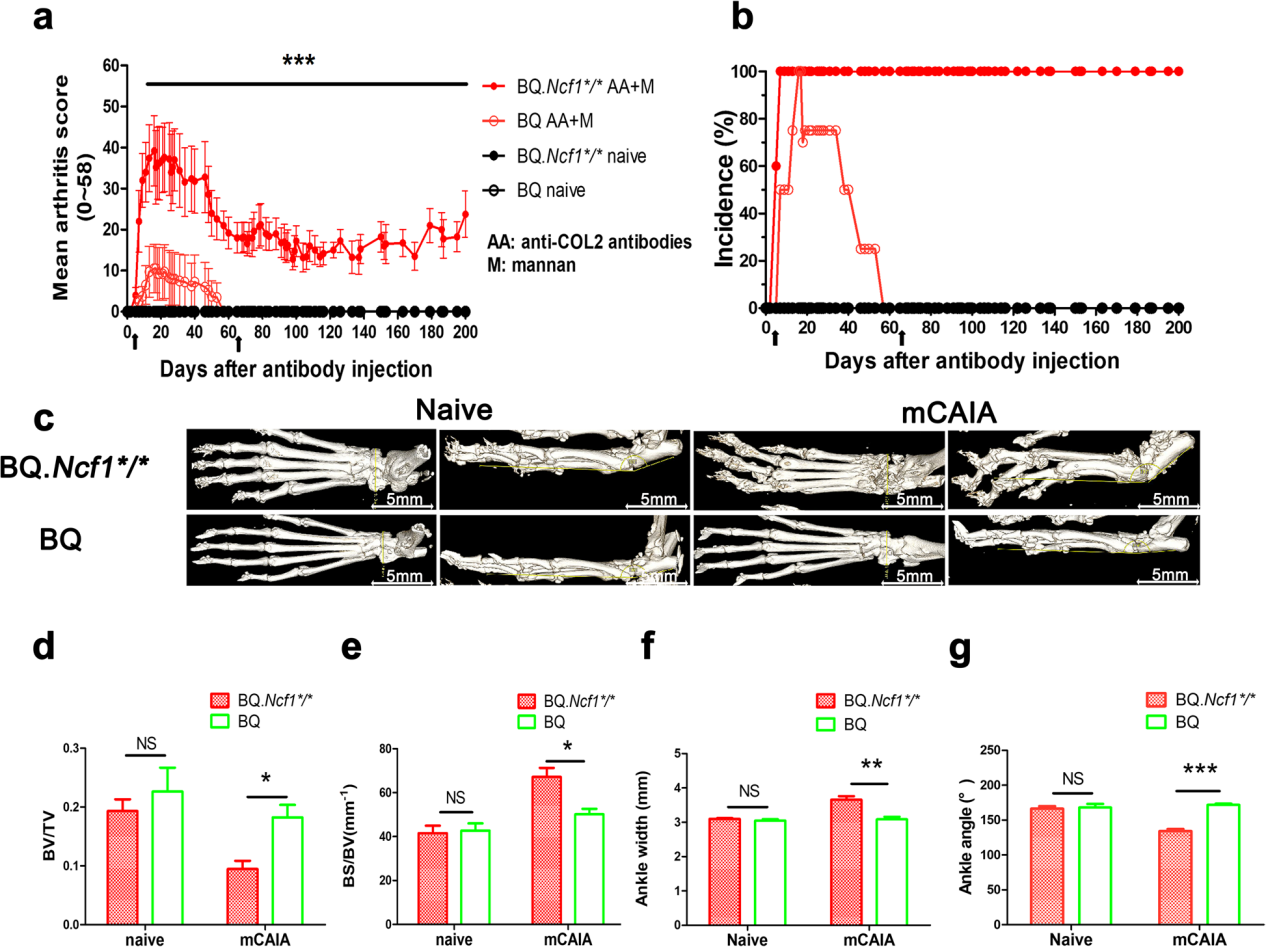
Bone damage in BQ.Ncf1*/* mouse paws during chronic arthritis. BQ.Ncf1^*/*^ mice and BQ mice in mCAIA group (14–16 weeks old, *n* = 4) and naïve group (*n* = 3) on day 200. **a** Arthritis score, (**b**) Disease incidence from day 100 to 200. **c** Representative images of mouse paws on day 200. Bone volume / Total volume (BV/TV) (**d**), Bone surface area / Bone volume (BS/BV) (**e**), ankle width (**f**) and ankle angle (**g**) in the distal region of the tibia were analyzed. Black arrows indicate i.p. injection of 4 mg of mannan each time per mouse, total 2 times. The data shown are mean ± SEM. The two-tailed Mann-Whitney U test was used to calculate the level of significance. BQ.Ncf1^*/*^ AA+M vs BQ AA+M: *, *p* <  0.05, **, *p* < 0.01, ***, *p* < 0.001

**Fig. 3:**
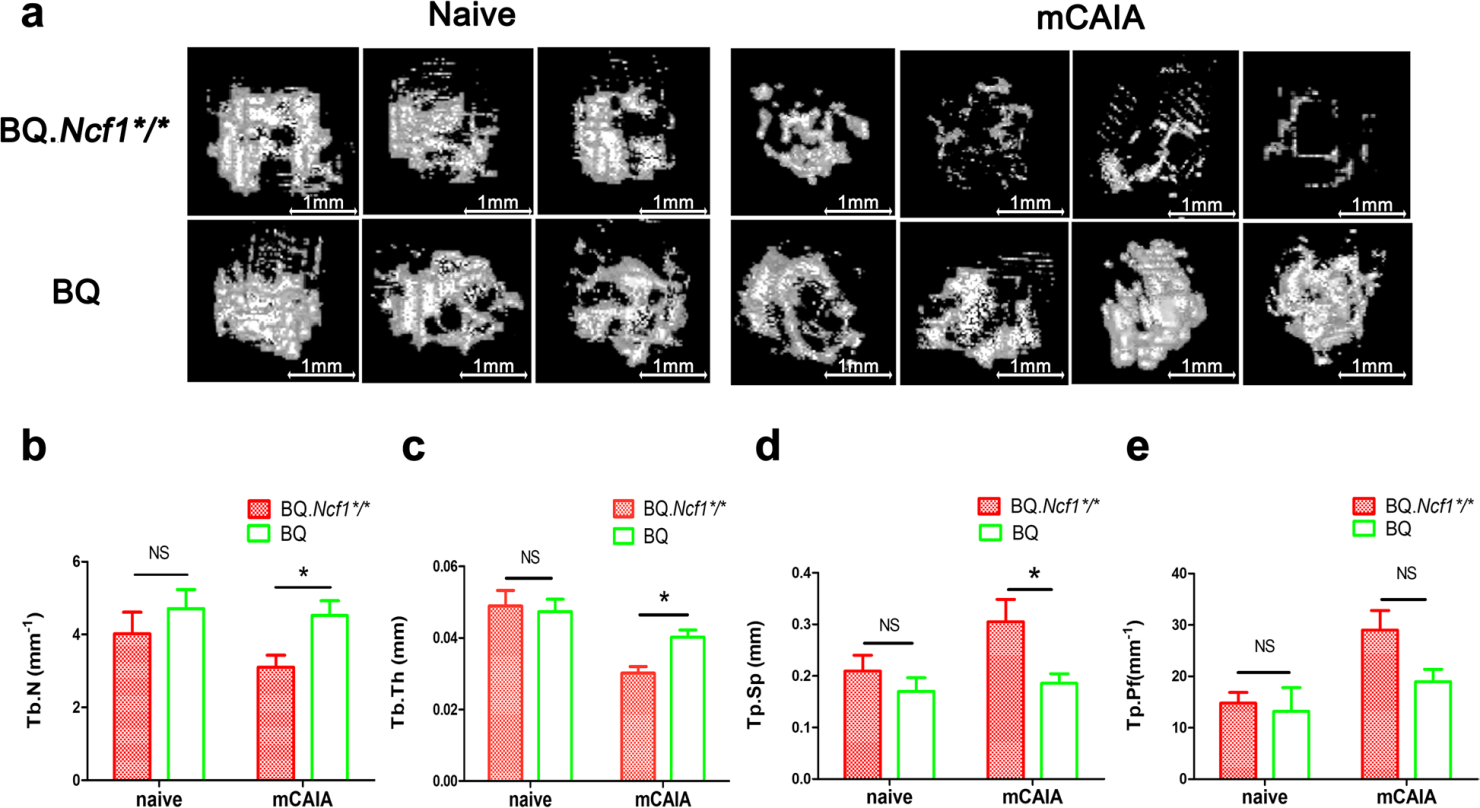
Distal tibial fractures damaged in the chronic phase of mCAIA in BQ.Ncf1^*/*^ mice. Micro-CT of BQ.Ncf1^*/*^ and BQ mCAIA (14–16 weeks old, *n* = 4) and naïve (*n* = 3) mice distal tibial fractures on day 200. **a** Micro-CT images of distal tibial fractures. **b** Trabecular number (Tb.N), (**c**) Trabecular thickness (Tb.Th), (**d**) Trabecular spacing (Tb.Sp), and (**e**) Trabecular pattern factor (Tb.Pf). The data shown are mean ± SEM. The two-tailed Mann-Whitney U test was used to calculate the level of significance. BQ.Ncf1^*/*^ AA+M vs BQ AA+M: *, *p* < 0.05, **, *p* < 0.01, ***, *p* < 0.001

Bone loss and joint destructions are characteristic features of the disease in a RA patient. We anticipated that similar phenomenon could be observed in the chronic phase of Ncf1^*/*^ mCAIA. Therefore, we observed the chronic phase of disease development for 200 days after the injection of antibodies and analyzed bone erosions using micro-CT [Fig. 2a-c]. Ratio of bone volume/total volume and ankle angle, ratio of bone surface area/bone volume and ankle width, trabecular number and thickness, trabecular spacing were measured and analyzed. The ratio of bone volume/total volume and ankle angle in BQ.Ncf1^*/*^ mice was lower than in the BQ mice [Fig. 2d and g]. Both the ratio of bone surface area/bone volume and ankle width of BQ.Ncf1*/* mice were higher than the BQ mice in the chronic phase [Fig. 2e and f]. In addition, the trabecular bone damage was more severe in the BQ.Ncf1*/* mice than in the BQ mice [Fig. 3a]. Trabecular number and thickness were decreased but trabecular spacing was increased in BQ.Ncf1^*/*^ mice compared to BQ mice [Fig. 3b-e]. These data showed that joint bones are eroded during the development of chronic arthritis in the BQ.Ncf1^*/*^ mice.

### Neutrophils and inflammatory cytokines increased in the chronic phase

**Fig. 4:**
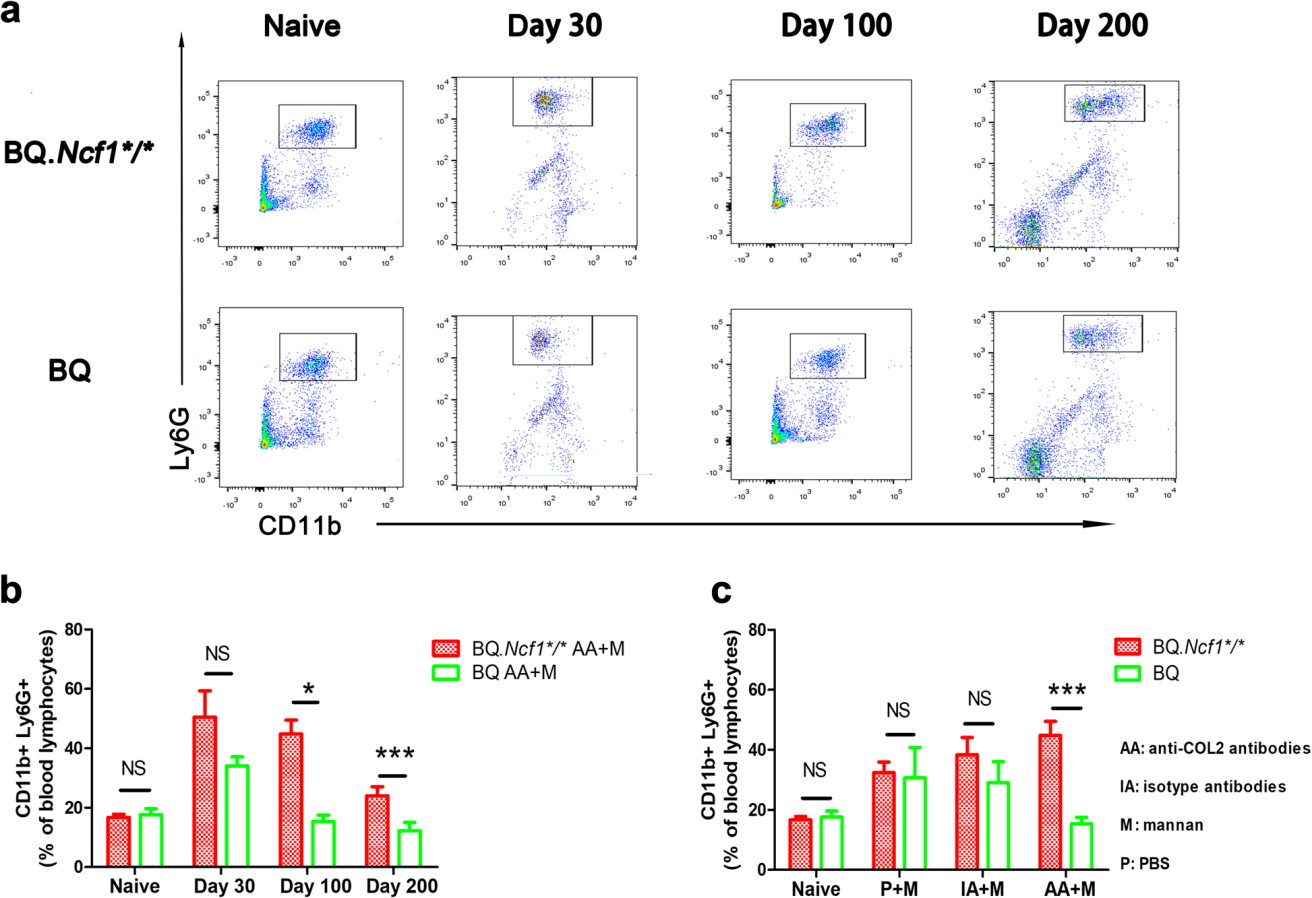
Increase of neutrophils in the chronic phase of mCAIA in BQ.Ncf1^*/*^ mice. FACS was performed using the blood lymphocytes on days 0 (naive) (*n* = 6–8), 30, 100 (*n* = 8–10) and 200 (*n* = 3–4). **a** Flow cytometry scatter plot. **b** Comparison of percentage of CD11b^+^Ly6G^+^ cells present in the blood between BQ.Ncf1^*/*^ mice and BQ mice on different days. **c** The percentage of CD11b^+^Ly6G^+^ cells present in the blood samples from different groups on day 100. The data shown are mean ± SEM. The two-tailed Mann-Whitney U test was used to calculate the level of significance. BQ.Ncf1^*/*^ AA+M vs BQ AA+M: *, *p* < 0.05, **, *p* < 0.01, ***, *p* < 0.001

**Fig. 5:**
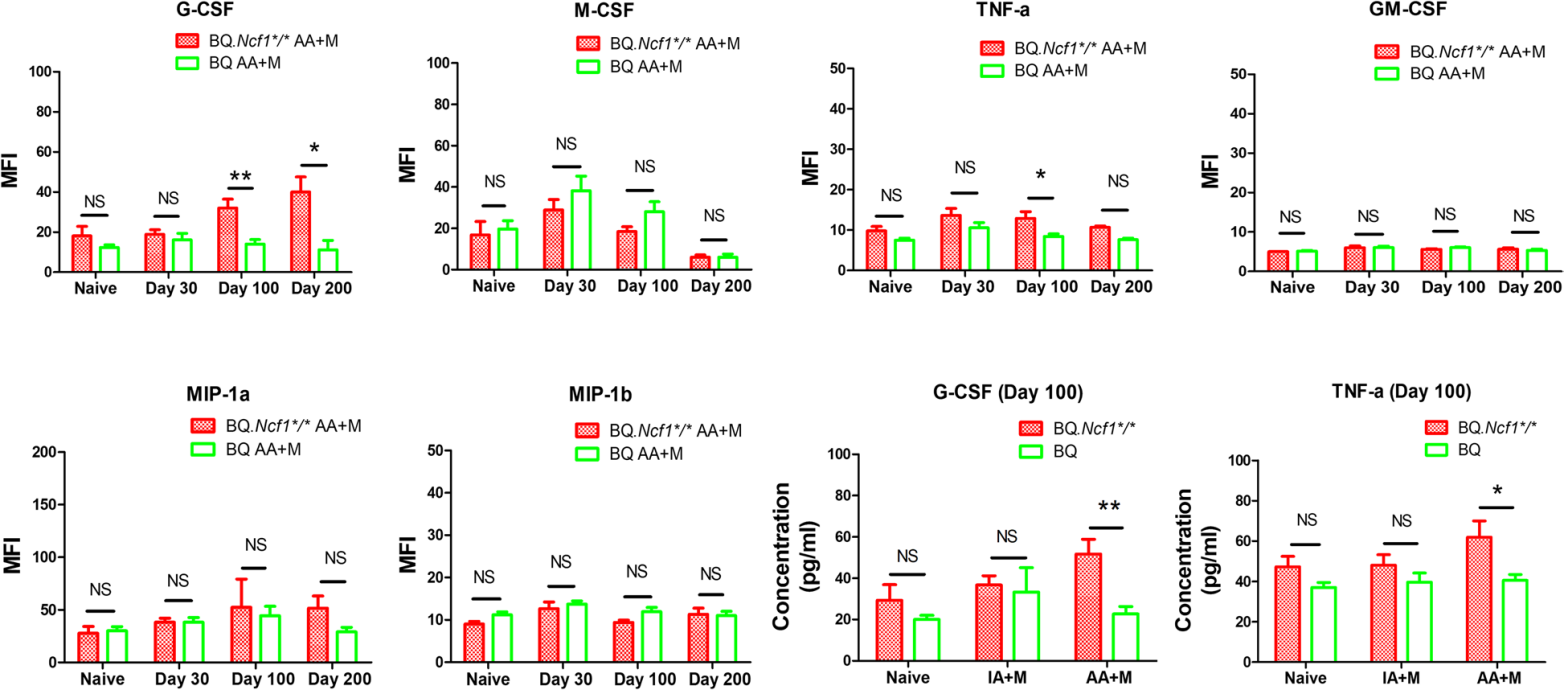
Levels of inflammatory cytokines in the chronic phase of mCAIA in BQ.Ncf1^*/*^ mice. Serum G-CSF, M-CSF, GM-CSF, TNF-α, MIP-1a, and MIP-1b in mCAIA on days 0 (naïve) (*n* = 5–6), 30 (*n* = 8), 100 (*n* = 8–10) and 200 (*n* = 3–4) and serum G-CSF and TNF-αon day 100 in AA + M (*n* = 8–9), IA + M (*n* = 4), P + M (*n* = 4) and naïve group (*n* = 6–8). (AA: anti-COL2 antibodies; IA: isotype antibodies; M: mannan; P: PBS). The data shown are mean ± SEM. The two-tailed Mann-Whitney U test was used to calculate the level of significance. BQ.Ncf1^*/*^ AA+M vs BQ AA+M: *, *p* < 0.05; **, *p* < 0.01; ***, *p* < 0.001

In RA, neutrophils are frequently occurring at the onset and occasionally also during the development of chronic arthritis [24]. Importantly, an increased level of neutrophils in blood were reported in chronic RA patients. We supposed that a similar increase in the level of neutrophils in blood could be observed in the chronic phase of mCAIA in BQ.Ncf1^*/*^mice. To clarify if the chronic arthritis was accompanied with a sustained activation of inflammatory cells, we analyzed the numbers of neutrophils (CD11b^+^Ly6G^+^ cells) in the blood from mCAIA mice. CD11b^+^Ly6G^+^ cells are considered as neutrophils in the mice [25, 26]. In the chronic phase (day 100–200) of mCAIA, the number of CD11b^+^Ly6G^+^ neutrophils in BQ.Ncf1^*/*^ mice was higher than in the BQ mice [Fig. 4a-c]. To confirm the increase of the systemic inflammatory responses during the chronic phase, we investigated the levels of inflammatory cytokines (G-CSF, M-CSF, GM-CSF, TNF-α, MIP-1a, and MIP-1b) in the blood. The concentration of G-CSF in sera was increased [Fig. 5], which agrees with the increased numbers of neutrophils reported earlier [27, 28]. We also observed an increase in TNF-α [Fig. 5], which is commonly seen during a chronic inflammation condition [29, 30]. Compared to the wild type mCAIA mice, which recovered from arthritis and had blood neutrophils returning to normal levels at day 100, BQ.Ncf1^*/*^ mCAIA had increased levels of blood neutrophils during the chronic phase. Along with this abnormal increase in neutrophils, a high level of G-CSF was observed in the chronic phase of mCAIA in BQ.Ncf1^*/*^. G-CSF, which can stimulate the survival, proliferation, differentiation, and function of neutrophil precursors and mature neutrophils, may be one of the main reasons for the observed abnormal increase in neutrophils during the chronic disease phase. However, the mechanisms behind the observed abnormal G-CSF level remains unclear.

### The injected anti-COL2 antibodies were detectable on day 100 but not on day 200

**Fig. 6:**
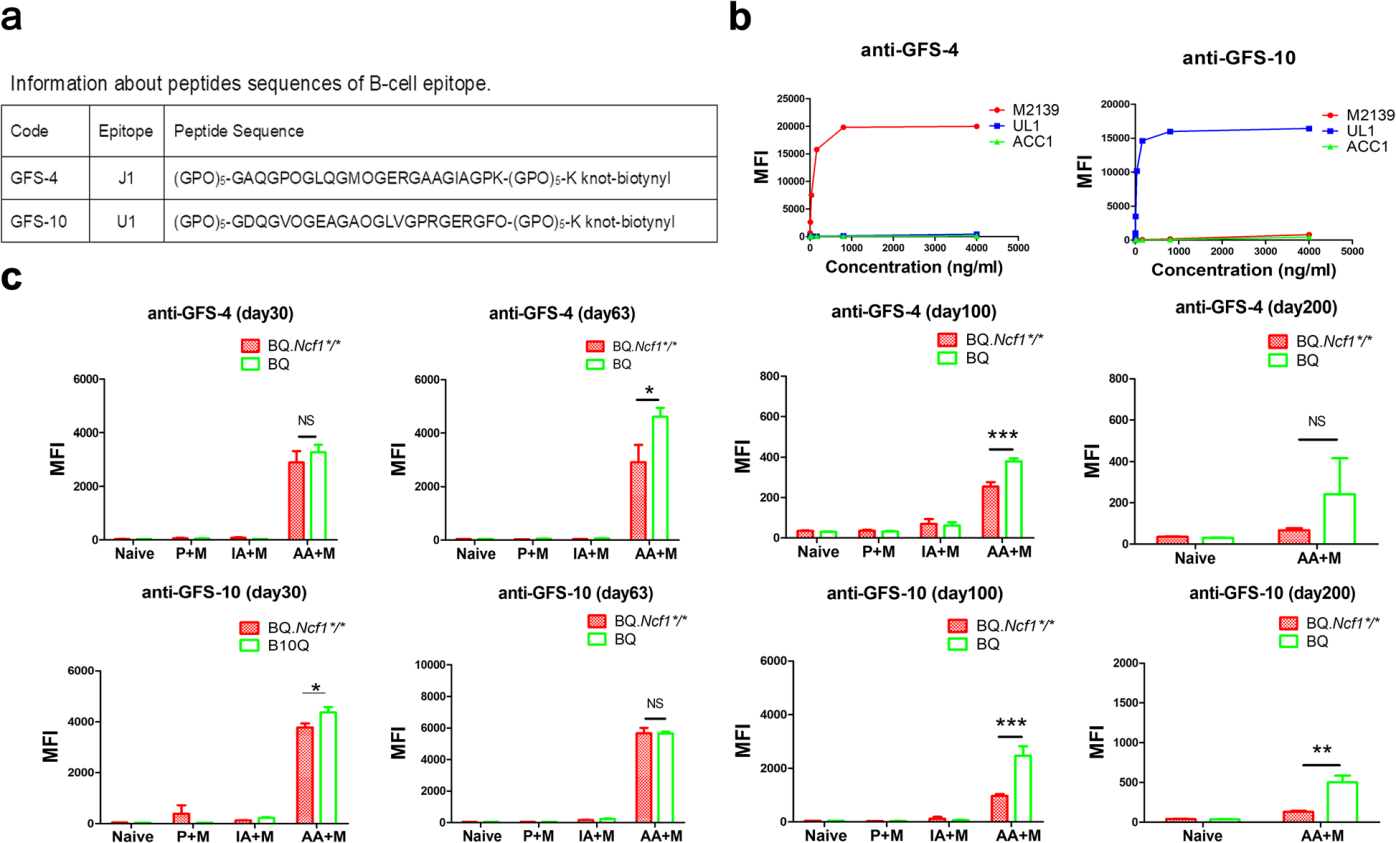
M2139 and UL1 antibodies are not detectable in the chronic phase of arthritis in BQ.Ncf1^*/*^ mice. The peptide sequence and the specificity of the injected antibodies (M2139 and UL1) are shown in (**a**) and (**b**), respectively. **c** The levels of M2139 and UL1 in the serum (1:50 dilution) from AA + M, IA + M, P + M and naïve groups on days 30, 63, 100 and 200. The data shown are mean ± SEM. The two-tailed Mann-Whitney U test was used to calculate the level of significance. BQ.Ncf1^*/*^ AA+M vs BQ AA+M: *, *p* < 0.05; **, *p* < 0.01; ***, *p* < 0.001

In our previous study, synthesis of new anti-COL2 antibodies was observed in CAIA model [16]. To understand whether anti-COL2 antibodies could contribute to the chronic phase of mCAIA in BQ.Ncf1^*/*^ mice, we measured the levels of the injected antibodies at days 30, 100, and 200 [Fig. 6, Table 2]. For the detection of specific antibodies, we used synthetic triple-helical peptides (J1 and U1 epitopes) [Fig. 6a] [31]. The specificity of the M2139 antibody to GFS-4 peptide and the UL1 antibody to GFS-10 peptide was observed [Fig. 6b]. During the active chronic arthritis period, J1 epitope specific M2139 antibody and U1 epitope specific UL1 antibody were still detectable at day 100, albeit at very low levels, but none of the antibodies were detectable at day 200 [Fig. 6c]. It seems that new anti-COL2 antibody generation in the chronic phase of mCAIA in BQNcf1^*/*^ mice is negligible. However, interestingly, in the corresponding wild type mice, with less chronic arthritis, the UL1 antibodies could still be detected at day 200, indicating that a functional *Ncf1*gene, or the absence of active arthritis, may be required for the longer half-life of injected antibodies.

**Table 2:** Blood M2139 and UL1 levels at different time points in mCAIA

	M2139			UL1		
	BQ.Ncf1*/* AA + M (ng/ml, ±SEM)	BQ AA + M (ng/ml, ±SEM)	***p***-Value	BQ.Ncf1*/* AA + M (ng/ml, ±SEM)	BQ AA + M (ng/ml, ±SEM)	***p***-Value
**Day 30**	**358.0 ± 54.39**	**407.6 ± 35.90**	**N.S.**	**345.0 ± 15.60**	**403.6 ± 20.65**	**N.S.**
**Day 63**	**361.9 ± 83.15**	**574.3 ± 43.00**	< 0.05 *	**531.8 ± 34.20**	**529.5 ± 11.61**	< 0.05 *
**Day 100**	**20.5 ± 2.94**	**35.0 ± 1.70**	< 0.01 **	**65.2 ± 6.457**	**216.0 ± 34.59**	< 0.001 ***
**Day 200**	**< 0**	**< 0**	**N.S.**	**< 0**	**20.2 ± 8.50**	< 0.01 **

*Notes: 1. M2139 concentration was calculated based on anti-GFS-4 antibody standard curve; 2. UL1 concentration was determined based on anti-GFS-10 antibody standard curve*

## Discussion

RA is a chronic and relapsing autoimmune disease but most disease models are mainly focused on the earlier preclinical stage of the disease, just before and after the onset of arthritis. We have earlier described a more relevant model for the chronic stage, which is induced with a combination of anti-COL2 antibodies and mannan in mice deficient for ROS production [16]. As shown in Fig. 1, we have successfully induced chronic mCAIA disease in the BQ.Ncf1^*/*^ mice but not in the wild type mice. Reduced level of ROS is an important factor in mannan-inducing mCAIA disease. The induced chronic arthritis lasted for at least 200 days, accompanied with a systemic inflammatory response associated with active joint erosions. Compared to the LPS-enhanced CAIA, which lasted for 20–30 days in general, mannan-enhanced CAIA in Ncf1^*/*^ mice was prolonged for longer period, closely reflecting the disease status of RA in humans. In Figs. 2 and 3, we evaluated the bone erosion parameters in the mCAIA using micro-CT and showed a reduction in ROS can enhance the trabecular bone loss. As depicted in Fig. 4, an abnormal increase in the level of neutrophils (CD11b + Ly6G+ cells) was observed in the chronic phase of mCAIA in BQ.Ncf1^*/*^ mice, similar to an increased neutrophils levels in the blood and synovial fluid from RA patients. Sustaining a high level of neutrophil levels may reflect the observed chronic inflammation in the mCAIA model. In Fig. 5, we explored the reason for such an increase in the level of neutrophils during chronic disease period. Interestingly, a high level of G-CSF was detected in the plasma samples from the Ncf1^*/*^ associated mCAIA during the chronic phase suggesting a high level of blood G-CSF may be contributing to the observed abnormal increase in neutrophils. In addition, we also detected an abnormal increase in TNF-α during chronic phase of mCAIA in BQ.Ncf1^*/*^ mice.

Chronic arthritis is known to develop independent of the adaptive immune system as it develops in mice deficient in B cells or αβ-T cells [16]. It is also independent of FcgRIII [16] and, as shown here, the injected arthritogenic anti-COL2 antibodies. Thus, active bone erosions during chronic arthritis can develop without B and T cells or autoantibodies, factors that are known to promote osteoclast activation and bone erosions [32]. Interestingly, chronic arthritis and bone erosions developed only in mice lacking the capacity to produce ROS, due to a profound deficiency in *Ncf1* functionality caused by one nucleotide mutation [16]. Importantly, polymorphism of the *Ncf1* gene, either through a single nucleotide polymorphism or by a copy number variation, has been shown to be a major genetic cause of autoimmune diseases such as systemic lupus erythematosus and RA [20] and similar to mutations in mice and rats, the mutation in the human *Ncf1* gene leads to NOX2 dysfunction. In SLE the Ncf1 gene is in fact the most important genetic factor but in RA the dominating genetic association are with the major histocompatibility complex region 2 (MHCII). In fact, the important role of MHCII alleles is shared with several animal models for RA, such as pristane induced arthritis and CIA [33, 34] [16]. MHCII codes for peptide receptors of key importance for the activation of antigen specific CD4^+^ T cells, a key interaction for causing specific antibody generation and believed to be important for the production of both antibodies to COL2 and ACPA [34–36]. However, our previous research has shown that induced arthritis was not different between mCAIA induced in the RAG-deficient Ncf1^*/*^ mice (with deficiency of the recombination activating gene, lacking B cells, αβ-T cells and natural killer T cells) and RAG-sufficient Ncf1^*/*^ mice [16]. It seems that *Ncf1* polymorphism can help induction of mCAIA without interaction with MHCII genes. More precisely, the causative role of the *Ncf1* mutation has been shown to be mediated by macrophages [16], or rather cells expressing CD68. This also involves macrophages developing into osteoclasts, but it remains to be investigated whether *Ncf1* deficient osteoclasts can independently be more highly activated, or rather, if ROS plays a regulatory role in macrophages to prohibit them from developing into osteoclasts. An alternative explanation could be that over activated, ROS-deficient, macrophages could activate a chronic stimulation of fibroblasts, a pathway that have been suggested to lead to chronic disease development in RA [37].

The pathogenic driving forces behind the chronic active arthritis in RA are still not known, an issue that is certainly difficult to address conclusively. More surprisingly there is also limited knowledge in animal models, mainly due to that chronicity in animal models are rarely addressed. It should be emphasized, however, that chronic development of arthritis could have several different driving forces. Apart from fibroblasts and macrophages discussed above there is no reason to exclude the involvement of the adaptive immune system in driving chronic arthritis in RA or in some animal models for RA [38]. In our previous study, we found no difference in the development of mCAIA between the RAG-deficient Ncf1^*/*^ mice and RAG-sufficient Ncf1^*/*^ mice [16]. It seems that the participation of the adaptive immune system is not necessary for the onset and duration of chronic phase of Ncf1^*/*^ mCAIA. However, only 4–6% of RA patients harbor the *Ncf1* mutation [16]. Moreover, RA has three stages, which are autoimmune priming in healthy individual stage, clinical onset stage and chronic inflammation stage respectively. How the inflammation turn into chronic phase is far from clear. Whether the adaptive immune system participate into the chronic phase remains unknown, there are many types of gene alterations suggested to be associated with RA, such as HLA-DRB1, PTPN22, AFF3, CD28, CD40, and CTLA4 [3].

Immunization of autologous COL2, instead of heterologous COL2 that is more often used, will lead to development of chronic arthritis in both mice and rats [39, 40], diseases that are clearly T cell dependent [41]. For example, in the pristane induced arthritis model in the rat, it is possible to transfer chronic relapsing arthritis with classical αβ-T cells [38]. Thus, there are several animal models now available to address different pathways leading to chronic relapsing arthritis and it is an open question as to which of these pathways lead to chronic arthritis in different subtypes of RA. The arthritis of mCAIA in the BQ.Ncf1^*/*^ mouse is a chronic and relapsing disease, which also includes a systemic inflammatory response and erosions of the affected joints. Thus, this model can be used to understand how innate immune mechanisms cause chronic and destructive joint damage. In this direction, more studies are needed to explore the contribution of G-CSF and neutrophil generated ROS in the chronic phase of mCAIA in Ncf1^*/*^ mice.

## Materials and methods

### Animals

BALB/c mice were from the Jackson Laboratories (Bar Harbor, ME, USA). B10.Q/rhd sub-strain, short-named as BQ, is under a standard genetic background whereas founders of B6/N mice were derived from Jackson Laboratories. B10Q.*Ncf1*^*m1j/m1j*^ (short-named as BQ.Ncf1^*/*^) has a mutation in the *Ncf1* gene [42]. In our experiments, BALB/c 10–12 weeks old mice were used to produce ascites used for purification of the needed antibodies. BQ. Ncf1^*/*^ 14–16 weeks old male mice and BQ 14–16 weeks old male mice were used at the onset of experiments. All mice were age-matched, randomized to experimental groups and cages, and evaluated by researchers blinded for the identity of mice. We bred and kept all the mice in Song Shan Lake Experimental Animal Science and Technology Park, Southern Medical University, Dongguan, China, with a specific pathogen-free, climate-controlled environment having a 14 h light/10 h dark cycles. The animals were housed in intra-cage ventilated polystyrene cages containing standard chow and water ad libitum with enrichments placed in the cages.

### Antibody production

B cell hybridomas secreting M2139 (anti-COL2 antibodies of IgG2b/κ subclass) [43], UL1 (anti-COL2 antibodies of IgG2b/κ subclass) [44], ACC1 (anti-COL2 antibodies of IgG2a/κ subclass) [45], G11 (mouse anti-human parathyroid epithelial cells of IgG2b/κ subclass) [46] and L243 (mouse anti-human HLA-DRα of IgG2a/κ subclass) (from ATCC) were used. They were cultured in DMEM (SH30022.01, Hyclone, USA) containing 10% fetal bovine serum (P30–1302, PAN-biotech, German) and 25 μg/mL of kanamycin (420311-25GM, Sigma, USA). Antibodies were generated by inducing ascites in mice [47]. 8–12 weeks old BALB/c mice were injected intraperitoneally (i.p.) with pristane (P2870-100ML, Sigma, USA), 0.5 mL per mouse. After two weeks, 0.5 mg of erythrocin (E6376-25G, Sigma, USA) was injected i.p. and 4 hours later with 1 × 10^7^ hybridoma cells. 10–14 days later, the mice were sacrificed by cervical dislocation and ascites was collected. The ascites fluid was centrifuged at 4000 rpm for 15 min and the sediment was discarded. Sodium azide (S2002-100G, Sigma, USA) was added to the supernatant, sterilized by filtration, and frozen at − 20 °C until used.

### Antibody purification

Antibodies were purified by ammonium sulfate precipitation [48]. (A) The ascites supernatant was defrosted, filtered, and the volume was measured. (B) Saturated ammonium sulfate (A4418-500G, Sigma, USA) was slowly added to the ascites supernatant to achieve a level of 55%, stirred for 30 min and left overnight at 4 °C to get the protein precipitate. (C) The protein suspension was centrifuged at 12000 rpm and 4 °C for 30 min. (D) The precipitate was dissolved in 10 mL PBS (10,010,023, Gibco BRL, Invitrogen AB, Sweden) with rigorous mixing, and when no obvious particles were found, the solution was filtered using 0.22 μm syringe filters (Dynagard, Spectrum Laboratories, CA, USA). (E) Sodium azide was added and the solution was stored at 4 °C. In the second step, the antibodies were purified by affinity chromatography with the ÄKTA system [49]. (A) A 10 mL column containing Gamma-bind plus affinity gel matrix (Gamma Bind Plus Sepharose; GE Healthcare, Uppsala, Sweden) was brought to room temperature (RT). (B) The following reagents were prepared for affinity purification: ultrapure water, 20% alcohol, elution buffer (0.1 M glycine•HCl, pH 2.7), column cleaning solution (5 M guanidine-HCl (1,042,190,500, Sigma, USA) solution), and the binding buffer (phosphate solution, pH 7.0). (C) The gel column was installed on the GE AKTA™ pure 25 protein purifier and washed with ultra-pure water to remove the alcohol used as a preservative. The parameters of the GE AKTATM pure 25 instrument were as follows: Pressure limitation = 0.3 mpa and flow rate = 3.0 mL/min. (D) The column was saturated with 50 mL binding buffer. (E) Protein samples were injected with a flow rate = 2.0 mL/min with a pressure limitation = 0.3 mpa. (F) Antibodies were eluted with a flow rate of elution buffer = 1.0 mL/min and a pressure limitation = 0.3 mpa. Antibodies were collected in tubes and a neutralization solution, Tris (pH 8.0), was used to adjust the pH of eluted fractions to neutral condition. The antibody solutions were concentrated with an Amicon® Ultra ultra-filter (UFC5050, Sigma, USA).

### Endotoxin removal and detection

ToxinEraser™ Endotoxin Removal Kits (L00338, Genscript, China) were used to remove the endotoxin and ToxinSensor™ Chromogenic LAL Endotoxin Assay Kits (L00350C, Genscript, China) were used to detect the endotoxin present in the antibody solutions and found to be lower than 1 EU/mL.

### Antibody quantification

The total antibody concentration was quantified by freeze-drying the samples, followed by measuring the weight of each sample. ELISA was used to quantify the level of antigen specific antibodies (M2139, UL1, and ACC1). (A) Rat collagen type II (COL2) was diluted to 5 μg/mL in PBS and added to 96-well ELISA plate (3361, Corning, USA), 50 μL for each well. The plate was sealed and placed at 4 °C overnight. (B) The plate was washed with PBS containing 0.05% tween-20 (93773-250G, Sigma, USA) (PBS-T) 4 times. (C) A blocking solution, 150 μL 3% skim milk (1,153,630,500, Sigma, USA) was added into each well and the plate was incubated for 2 h, at RT. (D) The plate was washed with PBS-T 4 times. (E) 50 μL of M2139, UL1, and ACC1 antibody standard solution or sample solution was added. (F) The plate was washed with PBS-T 4 times. (G) Human ads-HRP Goat anti-mouse IgG (ab97023, Abcam, UK) secondary antibody diluted in PBS at 1:400 was added and incubated for 40 min at RT, 50 μL per well. The plate was washed with PBS-T 4 times. (H) 50 μL of TMB (3,3′,5,5′-tetramethylbenzidine) solution (CL07-100ML, Sigma, USA) was added, and the OD value was measured at λ = 450 nm after 10 min at RT.

### Induction of arthritis

M2139, UL1, and ACC1 antibodies were mixed ar 1:1:1 ratio and diluted with PBS to obtain the anti-COL2 antibody mixture. G11 and L243 were mixed in at 2:1 ratio and diluted in PBS to obtain the isotype-control antibody mixture. BQ.Ncf1^*/*^ and BQ male mice (14–16 weeks) was injected i.v. with 9 mg of antibody mixture or PBS. Mannan (M7504, CAS Number: 9036-88-8, Sigma, USA) was diluted to 8 mg/mL and 4 mg was injected intraperitoneally for each mouse on days 3 and 65. Arthritis was scored in a double-blind manner using a previously described scoring system [50]. Each inflamed toe or finger joint got 1 point, whereas wrist and ankle joint inflammation received 5 points, so that each paw was given a maximum score of 14 points (4 phalangeal joints + 5 metacarpophalangeal joints + 1 wrist/ankle), and a mouse had a maximum score of 58.

### Micro-CT (micro-computed tomography)

The mice were sacrificed with cervical dislocation on day 200 after injection of antibodies, the hind paws were taken from 0.3–0.6 mm above the ankle joint, and the joint micro-CT (Siemens Inveon, Germany) scan was performed. The changes in bone structure were analyzed using CT analysis software. ROIs were plotted around the distal tibial joint. Bone volume/total bone volume ratio, bone surface area/bone volume ratio, trabecular number ratio, trabecular thickness, space between trabeculae, and trabecular model factors were evaluated.

### Bead-based multiplex immunoassays

Autoantibody responses were analyzed using a Luminex technology platform [51, 52]. Triple-helical peptides were synthesized using the Lys branching strategy [31, 53], characterized by mass spectrometry following trypsin digestion and circular dichroism spectroscopy [54, 55], and attached to magnetic beads as described earlier [56]. Peptide sequences correspond to the J1 epitope (GFS-4) and the U1 epitope (GFS-10). The serum samples were diluted at 1:50 (v/v) and the standard antibodies were diluted to 1 μg/mL with a buffer containing 100 μg/mL NeutrAvidin, 5% milk powder, 0.1% ProClin300, 3% BSA, 0.05% Tween-20 in PBS and incubated for 1 h with shaking at RT. The samples were transferred into the 96-well plate (Greiner Bio-One) containing peptide-coated magnetic beads and incubated for 75 min with shaking at RT. The beads were washed with PBS containing 0.05% Tween-20 (PBST) on a plate washer (EL406, Biotek or Bioplex Pro Wash station, Biorad). Goat anti-mouse IgG, Fcγ-PE (Jackson Immuno Research) was added as the secondary antibody onto the beads and incubated for 40 min with shaking at RT. The fluorescence intensity was measured by Luminex 200 (Luminex Corp.) as median fluorescence intensity (MFI) units.

### Luminex measurement for cytokines

The BQ.Ncf1^*/*^ and BQ mCAIA mice (*n* = 4–12) serum samples from days 30, 100, and 200 were used to measure concentrations of the cytokines G-CSF, M-CSF, GM-CSF, TNF-α, MIP-1a, and MIP-1b with the ProcartaPlex™ Simplex Kit (Thermofisher USA). The fluorescence intensity was measured by Luminex 200 (Luminex Corp.) as described earlier [51]. The MFI was used to quantify the level of cytokines.

### Flow cytometry (FCM) to detect blood CD11b + Ly6G+ cells

The peripheral blood from mice was collected and stored on ice in anticoagulant coated tubes. After centrifugation at 1600 rpm for 10 min, the cells were collected and added to a 15 mL centrifuge tube, and 5 mL Red Blood Cell Lysing Buffer Hybri-Max™ (R7757-100ML, Sigma, USA) was added to lyse erythrocytes, followed by centrifugation for 10 min. After 10 min, PBS (10 mL) was added to terminate the lysis process and the tube was centrifuged at 1600 rpm for 10 min. The precipitate was resuspended with 1 mL PBS and centrifuged at 1600 rpm for 5 min. The cells were resuspended in PBS to 1 × 10^7^–1 × 10^8^ cells/mL, incubated away from light for 30 min at RT with the FITC labelled CD11b antibody (#101206, clone M1/70, Biolegend, USA) and Alexa Fluor700 labelled Ly6G antibody (#127621, clone 1A8, Biolegend, USA) for surface marker staining, and centrifuged at 1600 rpm for 5 min at RT. The cells were washed with 500 μL of PBS 3 times and 300 μL of PBS was used to resuspend the cells. Analyses were performed using a BD Canto II flow cytometer (Becton-Dickinson Biosciences) and FlowJo software (Tree Star Inc., Ashland, OR, USA).

### Statistical analyses

Quantitative data were expressed as mean ± SEM using the GraphPad Prism version 7 software. For comparison of arthritis severity between BQ.Ncf1*/* AA+M and BQ AA+M, including arthritis score, bone and trabecular erosion parameters, level of blood neutrophils, antibodies and cytokines with Luminex, the two tailed Mann-Whitney test was used. *P*-values less than 0.05 were considered statistically significant. *, *p* <  0.05; **, *p* <  0.01; ***, *p* <  0.001.

## Data Availability

The datasets and analysis generated during the current study are available from the corresponding author upon reasonable request.

## Abbreviations

RA: Rheumatoid arthritis
RF: Rheumatoid factors
CAIA: Collagen type II antibody induced arthritis
*Ncf1*: Neutrophil cytosolic factor 1
mCAIA: Mannan-enhanced CAIA
NADPH: Nicotinamide adenine dinucleotide phosphate
NOX2: NADPH oxidase 2
ROS: Reactive oxygen species
FcγRIII: Fc receptor gamma III
COL2: Collagen type II
G-CSF: Granulocyte colony-stimulating factor
TNF-α: Tumor necrosis factor α
CT: Computed tomography
PBS: Phosphate buffered saline
MFI: Median fluorescence intensity
FCM: Flow cytometry
FITC: Fluorescein isothiocyanate
ELISA: Enzyme-linked immuno sorbent assay
M-CSF: Macrophage colony-stimulating factor
GM-CSF: Granulocyte-macrophage colony-stimulating factor
MIP-1a: Macrophage inflammatory protein-1a
MIP-1b: Macrophage inflammatory protein-1b
PE: Phycoerythrin
Ly6G: Lymphocyte antigen 6G precursor
AA: Anti-COL2 antibody
IA: Isotype antibody
